# Hypeprolactinemia: still an insidious diagnosis

**DOI:** 10.1007/s12020-020-02497-w

**Published:** 2020-09-19

**Authors:** Ludovica Aliberti, Irene Gagliardi, Romolo M. Dorizzi, Stefano Pizzicotti, Marta Bondanelli, Maria Chiara Zatelli, Maria Rosaria Ambrosio

**Affiliations:** 1 Department of Medical Sciences, Section of Endocrinology and Internal Medicine, University of Ferrara, Ferrara, Italy; 2 Clinical Pathology Unit, Hub Laboratory, Greater Area, Cesena, Italy; 3 Laboratory Division of the S. Anna Hospital, University of Ferrara, Ferrara, Italy

**Keywords:** Pitfalls, Hyperprolactinemia, Heterophile antibodies, Biotine

## Abstract

Hyperprolactinemia can have different causes: physiological, pharmacological, and pathological. When investigating the etiology of hyperprolactinemia, clinicians need to be aware of several conditions leading to misdiagnosis. The most popular pitfalls are: acute physical and psychological stress, macroprolactin, hook effect, even though antibodies interferences and biotine use have to be considered. A 52-year-old woman was referred to Endocrinology clinic for oligomenorrhoea and headache. She worked as a butcher. Hormonal evaluation showed very high PRL (305 ng/ml, reference interval: <24 ng/ml) measured with the ECLIA immunoassay analyzer Elecsys 170. The patient’s pituitary MRI was normal and macroprolactin was normal. Hormonal workup showed LH: 71.5 mU/ml (2–10.9 mU/ml), FSH: 111.4 mU/ml (3.9–8.8 mU/ml), Estradiol: 110.7 pg/mL (27–122 pg/ml). Since an interference was suspected, the sample was sent to another laboratory using a different assay. After antibody blocking tubes treatment (Heterophilic Blocking Tube, Scantibodies) PRL was 28.8 ng/ml (reference interval < 29.2 ng/ml). Analytical interference should be suspected when assay results are not consistent with the clinical picture. Endogenous antibodies (EA) include heterophile, human anti-animal, autoimmune and other nonspecific antibodies, and rheumatoid factors, that have structural similarities and can cross-react with the antibodies employed by the immunoassay, causing hyperprolactinemia misdiagnosis. The patient’s job (butcher), led us to suspect the presence of anti-animal antibodies. Clinicians should also carefully investigate the use of supplements. Biotin can falsely increase hormone concentration in competitive assays. Many clinicians are still not informed about these pitfalls that are not mentioned in some recent reviews on PRL measurement.

## Introduction

Hyperprolactinemia, the detection of serum prolactin (PRL) levels above the upper reference limit (commonly >20 ng/ml in men and 25 ng/ml in women) [1–3] can have different causes, physiological, pharmacological, and pathological (Table 1). The predominant physiological consequence of hyperprolactinemia is hypogonadotropic hypogonadism due to the suppression of GnRH pulsatility. Clinical manifestations vary according to age and sex of the patient and to the magnitude of PRL secretion increase. Clinical presentation in women with oligomenorrhea, amenorrhea, galactorrhea, decreased libido, infertility, and decreased bone mass is generally more clear and occurs earlier than in men [1–9]. The most common symptoms in men are erectile dysfunction, decreased libido, infertility, gynecomastia, decreased bone mass, while galactorrhea is rare [1–9].

**Table 1:** Main causes of hyperprolactinemia

Physiological	Pregnancy, breastfeeding, nipple stimulation, exercise, acute stress, venipuncture.
Pathological	Pituitary: prolactinoma, co-secreting GH-PRL adenoma, non-secreting adenoma, stalk effect from sellar/parasellar mass, empty sella, lymphocitic hyopophysitis, Rahtke’s cyst, irradiation, infiltrative disorders, head trauma.
	Systemic disease: renal failure, primary hypothyroidism, PCOS, cirrhosis, chest lesions.
Pharmacological	Antipsychotics/neuroleptics, antidepressants, antihypertensive, antiemetics, opioids.

Adapted from ref. [1]

Prolactinomas, that account for 25–30% of functioning pituitary tumors, are the most frequent cause of high PRL [1–9]. Prolactinomas can be microadenomas, more common in premenopausal women, and macroadenomas, more common in men and postmenopausal women [1–9]. Increased PRL concentration can also be induced by pituitary adenomas co-secreting GH and PRL and by sellar/parasellar masses causing stalk effect, as non-secreting adenomas [1–9]. When investigating the etiology of hyperprolactinemia, clinicians need to be aware of several conditions that can influence PRL measurement leading to misdiagnosis and, consequently, to inappropriate patient management. The pitfalls most frequently cited in papers and reviews are represented by acute physical and psychological stress, macroprolactin, hook effect.

Stress from any source (exercise, venipuncture, etc.) can lead to usually mild (<60 ng/ml) elevation of PRL concentration. In these cases a slightly elevated PRL should be confirmed at least once, repeating the sampling at 15–20-min intervals [1–9]. When a drug-induced rise in PRL is suspected, PRL sampling should be repeated after withdrawal of medications for at least 72 h, if possibile [8].

In healthy subjects and in prolactinomas, total circulating PRL comprises 65–85% monomeric 23-kDa PRL, 15–30% dimeric 40–60-kDa “big” PRL and <10% > 150 kDa “big-big PRL (or macroprolactin), usually composed of a complex formed by monomeric PRL and IgGs. Macroprolactin has a lower renal clearance and it is minimally active. Since macroprolactin is variably detected by the immunoassays currently used by laboratories, high PRL concentrations can be found in normally ovulating women and don’t require any treatment [1, 2]. The reference method for the detection of macroprolactin is size exclusion chromatography, but this technique is time consuming and expensive. Polyethylene glycol (PEG) acts as a “sponge,” which absorbs water of hydration from proteins, reducing their solubility and leading to their precipitation, and has been widely proposed and used as a screening method [5–9]. Many authors reported that the assays and the automated analyzers used by different laboratories differently recognize macroprolactin [10–12].

The “hook effect,” i.e., falsely normal or mildly elevated PRL while the true PRL concentration is many fold higher than the upper limit, can be found in presence of large pituitary macroadenomas (≥3 cm) and clinical manifestations typical of prolactinoma. When this situation is suspected, clinicians should carry out a serial dilution of serum sample to eliminate the artifact. Immunoassays are usually based on capture antibodies that are immobilized in a solid phase and a second antibody that is usually labeled with a chemiluminescent or a fluorescent signal [1–9]. These antibodies bind to the antigen (PRL) forming a “sandwich” and a signal with an intensity proportional to the concentration of PRL. The relative antigen-to-antibody proportion influences its interaction and may hamper the appropriate formation of the immunocomplexes. The hook effect occurs when extremely high PRL concentration saturates both the capture and the labeled antibody, preventing the formation of the “sandwich” and causing false-negative results [1–6, 13].

## Case report

A 52-year-old woman suffering from a 12 months oligomenorrhoea and headache was referred to Endocrinology clinic for investigations. The patient reported menarche at 14 years, two previous pregnancies, and normal menstrual cycles until 12 months before. Galactorrhea was not detected and gynecologic evaluation was normal. She worked as a butcher. Hormonal evaluation showed very high PRL (305 ng/ml, reference interval: <24 ng/ml) measured with the ECLIA immunoassay analyzer Elecsys 170 (Roche, Milan, Italy). This result was confirmed with the same assay in a sample collected on a different day avoiding venipuncture stress and repeating the sampling at 15–20 min intervals. Other laboratory exams showed LH: 71.5 mU/ml (2–10.9 mU/ml), FSH: 111.4 mU/ml (3.9–8.8 mU/ml), Estradiol: 110.7 pg/mL (27–122 pg/ml) while TSH, FT4, IGF1, GH were within the reference interval. The search for macroprolactin by PEG precipitation was negative. We excluded medication and supplements use, renal failure, and hypothyroidism as well as the other conditions known to cause hyperprolactinemia (breast stimulation, chest trauma, etc). The patient’s pituitary MRI was normal (no adenoma, no empty sella, no parasellar mass, etc). Since the presence of an interference was suspected, the sample was sent to another laboratory using a different immunoassay (DxI, Beckman, Milan): PRL concentration was 30.2 ng/ml (reference interval 3.3–26.7 ng/ml) and PEG precipitation research for macroprolactin was negative. Since clinical manifestations and neuroradiology imaging were not concordant with PRL concentration, we measured again PRL using a Centaur XP analyzer (Siemens, Milan, Italy) after antibody blocking tubes treatment (Heterophilic Blocking Tube, HBT, Scantibodies, Santee, CA, USA). Macroprolactin research was again negative while PRL after HBT treatment was 28.8 ng/ml (reference interval < 29.2 ng/ml).

## Discussion

Analytical interference should be suspected when assay results are not consistent with clinical picture. In the reported case, the presence of high gonadotropin levels (not consistent with central hypogonadism typical of true hyperprolactinemia), the absence of a pituitary adenoma, the difference in PRL values measured by the different employed analyzers and the patient’s job (butcher), led us to suspect the presence of anti-animal antibodies. These antibodies interfere with PRL measurement and can cause hyperprolactinemia misdiagnosis [14]. The currently available basic immunoassay formats for measuring hormones are two: the “sandwich” and competitive assay. Endogenous antibodies (EA) include heterophile, human anti-animal, autoimmune and other nonspecific antibodies, and rheumatoid factors that have structural similarities and can cross-react with the antibodies employed by the immunoassay causing erroneous results.

**Fig. 1:**
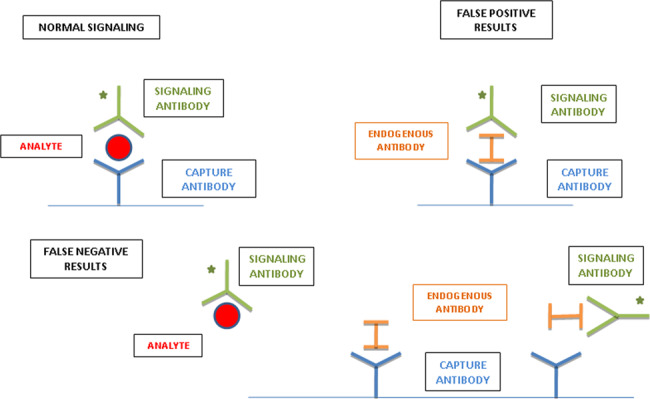
A representation of analyte and interfering endogenous antibodies (including heterophile antibodies) in a conventional two-site immunoassay, showing both false-positive and false-negative results

Heterophile antibodies, described as antibodies against red blood cell proteins of different species (e.g., rat, sheep, horse, rabbit, cow), are low avidity antibodies that occur naturally and do not require exposure to any immunogen [15–21]. Human anti-animal antibodies are high avidity and species-specific antibodies, produced following acute or chronic exposure to animal proteins. Circulating anti-animal antibodies can arise as a normal response of the human immune system to an administered “foreign” protein antigen [18–21]. Therapeutic administration of animal antisera and immunoglobulins (e.g., passive immunization with horse anti-tetanus antibodies), consumption of foodstuff (bovine milk and meat), prolonged exposure to animals (e.g., house pets) and animal products (e.g., meat treated by butchers) are the most common causes of the generation of specific human antibodies against animal immunoglobulins. Human anti-mouse antibodies are formed after the administration of diagnostic or therapeutic mouse monoclonal antibodies labeled with isotopes such as 99mTc or tagged with chemotherapeutic agents. Other sources of anti-animal protein are blood transfusion and vaccination, maternal transfer across the placenta to the unborn child, and the transfer of dietary antigens across the gut wall in diseases such as celiac disease [17–21]. The mechanism by which EA causes interference is different depending on the type of antibody and the immunoassay format. EA can lead to both falsely high and low analyte concentrations according to the site of interference (Fig. 1). EA usually cross-link capture antibodies with detection antibodies in the absence of antigen. Therefore, in this case, the system will detect the analyte (even if there is no analyte) and there will be a false-positive result. This type of interference is more common in sandwich assays. False-negative results are also possible: EA can reduce analyte concentration, especially in competitive assays. EA could cause interference for a number of analytes: macro-enzymes (creatine kinase, amylase), thyroid hormones (free and total forms), thyroglobulin, insulin, and testosterone [17].

Clinicians should also carefully investigate the use of supplements. In recent years many authors reported analytical interference of biotin in several immunoassays based on streptavidin-biotin capture techniques [22, 23]. Biotin is included in many over-the-counter multivitamins and used at very high concentration in biotinidase deficiency and multiple sclerosis, and in lower concentrations, but sufficient for interfering, for hair loss and brittle nails. In November 2017 the FDA released a safety warning that biotin supplementation may interfere in some laboratory assays. Biotin can falsely increase hormone concentration in competitive assays and decrease concentrations in sandwich assays [22–25]. However many clinicians are still not informed about this pitfall that is not mentioned in some recent reviews on PRL measurement [2, 7].

## Conclusion

In case of discrepancies between imaging, clinical picture, and the laboratory data, clinicians must consider the possibility of the presence of pre-, intra-, and post-analytical interferences. Recent reviews discussed many pitfalls in hyperprolactinemia diagnosis, such as venipuncture, macroprolactinemia, and hook effect. The present case report adds further pitfalls to be considered: EA interference and biotin. The development of more automated analyzers and communication between the requesting clinician and the laboratorian are essential to reduce the possibility that erroneous laboratory results cause harmful consequences to the patients.

## References

[1] Vilar L, Abucham J, Albuquerque JL, Araujo LA, Azevedo MF, Boguszewski CL (2018). Controversial issues in the management of hyperprolactinemia and prolactinomas—an overview by the Neuroendocrinology Department of the Brazilian Society of Endocrinology and Metabolism. Arch. Endocrinol. Metab..

[2] Vilar L, Vilar CF, Lyra R, Freitas MDC (2019). Pitfalls in the diagnostic evaluation of hyperprolactinemia. Neuroendocrinology.

[3] Melmed S, Casanueva FF, Hoffman AR, Kleinberg DL, Montori VM, Schlechte JA (2011). Diagnosis and treatment of hyperprolactinemia: an Endocrine Society clinical practice guideline. J. Clin. Endocrinol. Metab..

[4] Wang AT, Mullan RJ, Lane MA, Hazem A, Prasad C, Gathaiya NW (2012). Treatment of hyperprolactinemia: a systematic review and meta-analysis. Syst. Rev..

[5] Mancini T, Casanueva FF, Giustina A (2008). Hyperprolactinemia and prolactinomas. Endocrinol. Metab. Clin. North Am..

[6] Petersenn S, Giustina A (2020). Diagnosis and management of prolactinomas: current challenges. Pituitary.

[7] Petersenn S (2020). Biochemical diagnosis in prolactinomas:some caveats. Pituitary.

[8] Samperi I, Lithgow K, Karavitaki N (2019). Hyperprolactinaemia. J. Clin. Med..

[9] Saleem M, Martin H, Coates P (2018). Prolactin biology and laboratory measurement: an update on physiology and current analytical issues. Clin. Biochem. Rev..

[10] Kasum M, Oreskovic S, Zec I, Jezek D, Tomic V, Gall V (2012). Macroprolactinemia: new insights in hyperprolactinemia. Biochem. Med..

[11] Vieira JG, Tachibana TT, Ferrer CM, de Sá J, Biscolla RP, Hoff AO (2010). Hyperprolactinemia: new assay more specific for the monomeric form does not eliminate screening for macroprolactin with polyethylene glycol precipitation. Arq. Bras. Endocrinol. Metabol..

[12] Smith TP, Suliman AM, Fahie-Wilson MN, McKenna TJ (2002). Gross variability in the detection of prolactin in sera containing big big prolactin (macroprolactin) by commercial immunoassays. J. Clin. Endocrinol. Metab..

[13] Frieze TW, Mong DP, Koops MK (2002). “Hook effect” in prolactinomas: case report and review of literature. Endocr. Pract..

[14] Sapin R, Simon C (2001). False hyperprolactinemia corrected by the use of heterophilic antibody-blocking agent. Clin. Chem..

[15] García-González E, Aramendía M, Álvarez-Ballano D, Trincado P, Rello L (2015). Serum sample containing endogenous antibodies interfering with multiple hormone immunoassays. Laboratory strategies to detect interference. Pract. Lab. Med..

[16] Dodig S (2009). Interferences in quantitative immunochemical methods. Biochem. Med..

[17] Tate J, Ward G (2004). Interferences in immunoassay. Clin. Biochem. Rev..

[18] Mongolu S, Armston AE, Mozley E, Nasruddin A (2016). Heterophilic antibody interference affecting multiple hormone assays: Is it due to rheumatoid factor?. Scand. J. Clin. Lab. Invest..

[19] Bolstad N, Warren DJ, Nustad K (2013). Heterophilic antibody interference in immunometric assays. Best. Pract. Res. Clin. Endocrinol. Metab..

[20] Sturgeon CM, Viljoen A (2011). Analytical error and interference in immunoassay: minimizing risk. Ann. Clin. Biochem..

[21] Haddad RA, Giacherio D, Barkan AL (2019). Interpretation of common endocrine laboratory tests: technical pitfalls, their mechanisms and practical considerations. Clin. Diabetes Endocrinol..

[22] Li D, Ferguson A, Cervinski MA, Lynch KL, Kyle PB (2020). AACC guidance document on biotin interference in laboratory tests. J. Appl. Lab. Med..

[23] Dorizzi RM (2017). Biotin and interferences in immunoassays; problems and opportunities. Riv. Ital. Med. Lab..

[24] Lau CS, Aw TC (2019). A current approach to hyperprolactinemia. Int. Arch. Endocrinol. Clin. Res..

[25] Li D, Radulescu A, Shrestha RT, Root M, Karger AB, Killeen AA (2017). Association of biotin ingestion with performance of hormone and nonhormone assays in healthy adults. JAMA.

